# Job insecurity and health: A study of 16 European countries

**DOI:** 10.1016/j.socscimed.2009.11.022

**Published:** 2010-03

**Authors:** Krisztina D. László, Hynek Pikhart, Mária S. Kopp, Martin Bobak, Andrzej Pajak, Sofia Malyutina, Gyöngyvér Salavecz, Michael Marmot

**Affiliations:** a Institute of Behavioural Sciences, Semmelweis University, Budapest, Hungary; b Preventive Medicine, Department of Public Health Sciences, Karolinska Institute, Stockholm, Sweden; c International Institute for Society and Health, Department of Epidemiology and Public Health, University College London, London, UK; d Department of Epidemiology and Population Studies, Jagiellonian University, Krakow, Poland; e Institute of Internal Medicine, Russian Academy of Medical Sciences, Novosibirsk, Russia

**Keywords:** Europe, Job insecurity, Self-rated health, Effect modification

## Abstract

Although the number of insecure jobs has increased considerably over the recent decades, relatively little is known about the health consequences of job insecurity, their international pattern, and factors that may modify them. In this paper, we investigated the association between job insecurity and self-rated health, and whether the relationship differs by country or individual-level characteristics. Cross-sectional data from 3 population-based studies on job insecurity, self-rated health, demographic, socioeconomic, work-related and behavioural factors and lifetime chronic diseases in 23,245 working subjects aged 45–70 years from 16 European countries were analysed using logistic regression and meta-analysis. In fully adjusted models, job insecurity was significantly associated with an increased risk of poor health in the Czech Republic, Denmark, Germany, Greece, Hungary, Israel, the Netherlands, Poland and Russia, with odds ratios ranging between 1.3 and 2.0. Similar, but not significant, associations were observed in Austria, France, Italy, Spain and Switzerland. We found no effect of job insecurity in Belgium and Sweden. In the pooled data, the odds ratio of poor health by job insecurity was 1.39. The association between job insecurity and health did not differ significantly by age, sex, education, and marital status. Persons with insecure jobs were at an increased risk of poor health in most of the countries included in the analysis. Given these results and trends towards increasing frequency of insecure jobs, attention needs to be paid to the public health consequences of job insecurity.

## Introduction

As a result of globalisation, deregulation of labour markets and increasing competition, many companies worldwide have been forced during the last decades to undertake restructuring, downsizing and mergers and to introduce temporary or short term contracts. Although these events have a reasonable managerial rationale, they are perceived as threatening by the employees affected by these decisions, create insecurity and undermine the confidence in the company. They influence negatively attitudes towards the job and the organisation (Sverke, Hellgren, & Näswall, 2002), reduce productivity and increase costs for the society.

Beside their effects on organizational functioning, insecure jobs are also known to detrimentally affect employees' health (Ferrie, 2001). Low job security has been repeatedly found to be related to somatic (Ferrie, Shipley, Marmot, Stansfeld, & Smith, 1998a, 1998b; Ferrie, Shipley, Stansfeld, & Marmot, 2002; Mohren, Swaen, van Amelsvoort, Borm, & Galama, 2003) and minor psychiatric morbidity (D'Souza, Strazdins, Lim, Broom, & Rodgers, 2003; Ferrie et al., 1998a; Ferrie, Shipley, Newman, Stansfeld, & Marmot, 2005; Pelfrene et al., 2003; Rugulies, Bültmann, Aust, & Burr, 2006; Swaen, Bultmann, Kant, & van Amelsvoort, 2004), to poor self-rated health (Cheng, Chen, Chen, & Chiang, 2005; D'Souza et al., 2003; Ferrie, Shipley, Marmot, Stansfeld, & Smith, 1995; Ferrie et al., 1998a, 2005), as well as to incident coronary heart disease (Lee, Colditz, Berkman, & Kawachi, 2004) and its risk factors, including high cholesterol (Ferrie et al., 1995), hypertension (Ferrie et al., 1995, 1998b; Levenstein, Smith, & Kaplan, 2001) and obesity (Ferrie et al., 2002). Other, less direct measures of the health status, such as sickness absence (Kivimaki et al., 1997) and health services use (Roskies & Louis-Guerin, 1990) have also been found to be associated with job insecurity.

Despite the important results regarding the effect of job insecurity on health, the question of international differences in the health consequences of job insecurity have not yet been investigated (Erlinghagen, 2008). Most of the studies focusing on the association between job insecurity and health have been conducted in a few wealthy countries, primarily in the United Kingdom (Ferrie et al., 1995, 2005), Nordic countries (Lau & Knardahl, 2008; Rugulies, Aust, Burr, & Bültmann, 2008), Belgium (Pelfrene et al., 2003), United States (Lee et al., 2004) and Australia (D'Souza et al., 2003). Little is known about the effect of job insecurity in other European countries, especially in Central and East European countries which have been particularly stricken by this problem during the period of their transition from a centralised to a market economy. Countries differ in the extent of their labour market regulations, their social security system, their health care, the degree of unionization and collective power and the population's experience of and coping with spells of unemployment. These factors may potentially act as buffers or may further increase the risk of illness due to job insecurity, contributing to between-country differences in the health consequences of job insecurity.

Furthermore, research regarding which groups within different countries are particularly vulnerable to the negative consequences of job insecurity is sparse and findings in this area are conflicting. First, it seems that individuals with low education are more negatively affected by job insecurity than those better educated given their poorer social and financial resources (Cheng et al., 2005; Sverke et al., 2002). However, Schaufeli (1992) suggests that highly educated individuals would suffer from “status inconsistency” when faced with job loss, inconsistency which would further increase strain and the risk of ill health.

Second, as women have higher temporary employment rates than men and suffer from discrimination in the labour market (Menéndez, Benach, Muntaner, Amable, & O'Campo, 2007; Munoz de Bustillo Lorente & de Pedraza, 2007), female employees may be more likely to be affected by job insecurity and its negative consequences than men (Menéndez et al., 2007). Others, however, have suggested that due to the greater social expectation of the work role for men (Cheng et al., 2005) and the protection provided for women by their alternative roles, the experience of job insecurity is actually more distressing for men than for women (De Witte, 1999; Ferrie et al., 1995).

Third, people at the middle of their working life might face a particularly high risk of health deterioration when experiencing job insecurity. The unemployment role is generally less acceptable for employees aged 30–50 years than for other age groups, due to their family responsibilities, bank loans and thus a strong dependency on a steady income (De Witte, 1999; Sverke et al., 2002). Unemployment at other ages might be less detrimental as young individuals would maintain their “youth role” for a little longer, whereas older persons might consider early retirement (De Witte, 1999; Sverke et al., 2002).

Finally, job insecurity may have more deleterious effects for single persons than for married or cohabiting ones, as the social and the financial support from a spouse is likely to have an important protective effect.

Therefore, the objective of the present study was to investigate whether job insecurity is associated with self-rated health, whether the association is similar in different European countries, and whether it is modified by socio-demographic factors.

## Methods

### Study populations

Data from three population-based studies, including participants from 16 European countries were analysed in the present study.

#### The Health, Alcohol and Psychosocial Factors in Eastern Europe (HAPIEE) study

The HAPIEE study consists of three cohorts recruited in Russia (Novosibirsk), Poland (Krakow) and Czech Republic (Havirov/Karvina, Hradec Kralove, Jihlava, Kromeriz, Liberec and Usti nad Labem). The study was described in detail elsewhere (Peasey et al., 2006). Briefly, the cohorts consist of random samples of men and women aged 45–70 years, stratified by gender and age, and selected from population registers. Data collection took place between 2002 and 2005. A total of 28,947 individuals completed the questionnaire with an overall response rate of 59%. Approximately 50% of the sample (*n* = 13,271) was in employment and answered a questionnaire module about work characteristics. The study received ethical approval from the UCL/UCLH joint research ethics committee and from ethical committees in each participating country and all participants gave informed consent.

#### The Survey of Health, Ageing and Retirement in Europe (SHARE)

Based on probability samples in all participating countries, SHARE represents the non-institutionalized population aged 50 and older from Austria, Belgium, Denmark, France, Germany, Greece, Israel, Italy, the Netherlands, Spain, Sweden and Switzerland (Boersch-Supan & Juerges, 2005). Spouses were also interviewed independently of their age, thus persons younger than 50 years were also included in the study. Data for the first wave of this longitudinal study were collected during 2004 in all countries, except Israel where data collection took place between 2005 and 2006. A total of 31,115 individuals from 21,176 households were interviewed. The average response for individuals was 85.3%. To allow comparability with the HAPIEE study, analysis for the present study were restricted to the working population aged 45–70 years participating in the first wave of the study (*n* = 8688).

#### The Hungarostudy Epidemiological Panel (HEP) 2006

The HEP 2006 is the second phase of the Hungarostudy 2002, a nation-wide representative survey of the adult population of Hungary (Susánszky et al., 2007). The sampling frame was the National Population Register. Towns with a population of more then 10,000, as well as a random sample of smaller settlements were included in the sample. Of the 8008 subjects who gave consent to participate in a follow-up study a total of 7321 persons (91.5%) could be traced in 2006. Of these subjects 318 (4.34%) were deceased, 1738 (23.73%) refused to participate in the study and 741 (10.12%) were not in adequate condition to complete the interview (e.g. due to illness, drunkenness), resulting in 4524 subjects being finally interviewed (Susánszky et al., 2007). Analysis for the present study were restricted to the working population aged 45–70 years from the follow-up sample (*n* = 1286). The study was approved by the Ethics Committee of the Semmelweis University in Budapest.

### Measurements

#### Job insecurity

Job insecurity in the HAPIEE study and in the HEP 2006 was assessed by means of the question “Is your own job security poor?” from the Effort-Reward Imbalance questionnaire (Siegrist, 1996). The answer to this question consists of two parts. First, respondents answer whether they are exposed to poor job security and if yes they indicate to what extent this is a source of distress for them. Answers are given on a 5-point scale: (1) no, (2) yes, I am not at all distressed, (3) yes, I am somewhat distressed, (4) yes, I am distressed, (5) yes, I am very distressed. For the present analysis the variable was dichotomized as without (alternative 1) or with job insecurity (alternatives 2–5).

The SHARE participants were asked to indicate on a 4-point Likert scale to what extent they agree with the statement ‘My job security is poor’. Those responding “strongly agree” and “agree” were regarded as having job insecurity.

#### Self-rated health

In all 3 studies participants were asked to rate their overall health as very good, good, fair, poor or very poor. The first two answer categories were considered as good health, whereas the “fair”, “poor” and “very poor” answers were classified as poor health.

#### Covariates

Educational attainment was classified into three levels: less than high school, completion of high school and college/university. Occupational status was categorized as managerial vs. non-managerial. Study participants were categorized as having a part-time job (on average up to 6 h of work/day), having a full-time job (on average 6–8 work hours/day) or working excess hours (on average more than 8 h of work/day) on the basis of the average number of work hours per week (in the HAPIEE study and in the SHARE) or per day (in the HEP 2006). Individuals were classified according to marital status as being single, married, cohabiting, divorced/separated or widowed. Age and data on lifetime medical diagnosis of coronary heart disease, stroke, hypertension, cancer or diabetes were also registered. Body-mass index (BMI) was calculated using recorded weight and height. Smoking was categorized as never, former or current smoker. Frequency of alcohol consumption was categorized as never, rarely or often. Those engaging less than 1 h per week (in the HAPIEE study) or less than once a week (in the other two studies) in physical activity were considered to be physically inactive. In the HAPIEE study this variable referred to sports, games, hiking and physically demanding activities such as housework, gardening, maintenance of the house. In the HEP 2006 the frequency of sport activities, e.g. swimming, jogging, cycling, playing football, aerobic and of other non-sport activities such as gardening, construction was measured. In the SHARE the variable referred to sports, heavy housework, or a job that involves physical labour and to activities such as gardening, cleaning the car, or going for a walk.

### Statistical analysis

Multiple logistic regression models were constructed for each of the 16 countries to analyze the association between job insecurity and poor health. Several models were constructed: 1) age- and sex-adjusted, 2) adjusted for age, sex, socioeconomic and work-related factors (education, managerial status, type of work, marital status), 3) adjusted for age, sex, socioeconomic, work-related factors and health behaviours (smoking, physical activity, BMI and alcohol consumption frequency) and 4) adjusted for age, sex, socioeconomic, work-related factors, health behaviour and lifetime diagnosis of coronary heart disease, stroke, hypertension, cancer or diabetes. Stratified analysis and formal tests for interaction were conducted to assess possible effect modification by age (split at 55 years), sex, education and marital status (living in a partnership vs. single). The analyses were conducted using SPSS 14.0 for Windows and Stata 10. Due to concerns about data sharing in some studies, individual-level data could not be combined into one dataset. However, we collectively could analyze all individual datasets used in this paper; to investigate the association between job insecurity and health in all 16 countries, country-specific odds ratios were pooled together using meta-analysis procedures in Stata 10. The heterogeneity of country-specific odds ratios (OR) was tested using the *Q* test statistic and the *I*^2^ measure of heterogeneity (Higgins, Thompson, Deeks, & Altman, 2003).

## Results

Table 1 presents the distribution of age, gender, job insecurity and self-rated health in the 16 samples included in our analysis. The percentage of male workers ranged from 42.6% (in Hungary) to 62.9% (in Greece). The prevalence of job insecurity was the lowest in Spain (14.2%) and France (17.6%) and the highest in Hungary (40.4%), Czech Republic (41.0%) and Poland (41.7%). The prevalence of fair, poor or very poor health was higher in the Hungarian, Czech, Russian and Polish samples compared to the SHARE countries.

Table 2 shows for each country separately the results of the multi-adjusted logistic regression analysis, conducted to investigate the association between job insecurity and self-rated health. In age- and sex-adjusted analysis job insecurity was significantly associated with an increased risk of poor health in the Czech Republic, Denmark, Germany, Hungary, Israel, the Netherlands and Poland. Statistically not significant results but with similar ORs were found in Austria, France, Greece, Italy, Spain and Switzerland. In Belgium, Russia and Sweden the observed association was weak. When performing alternative models we found no evidence for interaction between gender and age on self-rated health. Adding this interaction term to the model resulted in virtually identical effects of job insecurity on our outcome.

**Fig. 1:**
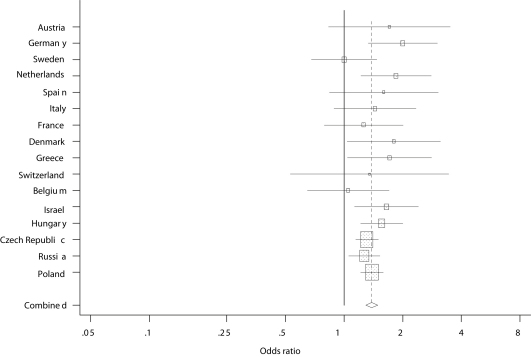
Country-specific and pooled OR (95% CI) for the association between job insecurity and self-rated health.

Further adjustment for socioeconomic and work-related factors, health behaviours and lifetime diagnosis of chronic diseases did not change the results considerably (Model 4). In the Czech Republic, Denmark, Germany, Greece, Hungary, Israel, the Netherlands, Poland and Russia job insecurity was positively and significantly associated with poor self-rated health. The ORs ranged between 1.27 (Russia) and 2.00 (Germany). Comparably strong, but statistically not significant associations were observed in France and in several countries with smaller sample sizes (Austria, Italy Spain and Switzerland). In Belgium and Sweden there was no association between job insecurity and health. Fig. 1 presents the country-specific and the pooled OR and 95% confidence interval (CI) from the fully adjusted model. Country-specific ORs did not differ significantly; the *Q* test statistic was 14.478 (df = 15, *p* = 0.49). The *I*^2^ measure of heterogeneity was 0, further supporting the homogeneity of the observed country-specific ORs. The average estimate was that job insecurity was associated with a 39% increased risk (95% CI: 1.30–1.49) of poor health in the overall sample.

**Fig. 2:**
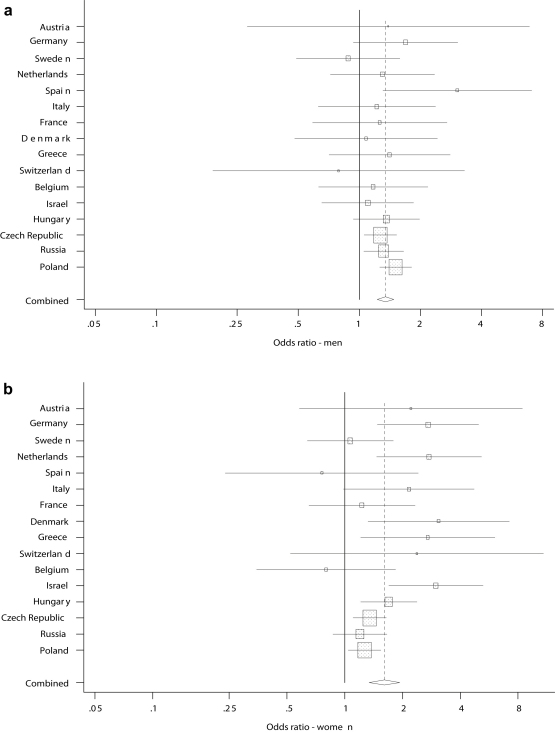
(a) Country-specific and pooled OR (95% CI) for the association between job insecurity and self-rated health for men. (b) Country-specific and pooled OR (95% CI) for the association between job insecurity and self-rated health for women.

We found roughly similar or not consistently different associations between job insecurity and perceived health in the subgroups based on age (split at 55 years), education, and marital status, indicating no effect modification from these variables. Fig. 2a and b present the country-specific and the pooled ORs and 95% CIs for the association between job insecurity and poor health from the fully adjusted models separately for the two genders. Compared to men, women had somewhat higher risk of poor health associated with job insecurity in Denmark, Germany, Greece, Israel, Italy and the Netherlands, whereas the risk of poor health due to job insecurity was somewhat higher in Spanish men compared to Spanish women. However, the sample sizes were generally too small to detect significant interaction effects. Only in Denmark and Israel was the interaction term between gender and job insecurity significant. In the pooled analysis the ORs (95% CIs) for the association between job insecurity and poor health were 1.61 (1.34–1.93) for women and 1.35 (1.23–1.48) for men, respectively. However, the interaction between gender and job insecurity was not significant (*p* = 0.20).

## Discussion

In this multi-country study, job insecurity was associated with an increased risk of poor health in most of the countries included in the analysis. The strength of the relationship between job insecurity and health did not differ according to age, sex, education, and marital status.

Our results are similar to findings from studies conducted in the United Kingdom (Ferrie et al., 2002, 2005), Australia (D'Souza et al., 2003), Nordic countries (Lau & Knardahl, 2008; Rugulies et al., 2008), Canada (McDonough, 2000) or Taiwan (Cheng et al., 2005) which document consistently the detrimental effects of job insecurity on general health. For example, Ferrie et al. (1995, 2002) found in two prospective studies that British civil servants facing their company's privatisation or who at an initial measurement reported having an insecure job were more likely to experience a poorer health at a later stage of the study. A prospective Danish study (Rugulies et al., 2008) found that employees in insecure jobs were at an increased risk to experience a decline in their self-rated health at a 4-year follow-up. Studies using cross-sectional data (Cheng et al., 2005; D'Souza et al., 2003; Pelfrene et al., 2003) and studies investigating other health measures documented similar relationships. The small effect of job security in our study is consistent with the modest effects reported by Sverke et al. (2002) in their meta-analysis.

A high proportion of the working European population aged 45–70 years perceive their jobs as insecure; the percentage of individuals within the sample of their countries reporting to have an insecure job ranged from 14.2% in Spain to 41.7% in Poland. Income from work makes up to 70% of the average family income in Europe, thus job insecurity has an important impact on life security as a whole (Munoz de Bustillo Lorente & de Pedraza, 2007). Job insecurity has been found to be one of the most important work-related stressor (De Witte, 1999), whereas job security was considered to be the most valued characteristics of jobs in all European countries – except for Denmark, the Netherlands and Sweden where it was ranked as the second most important after good relationships with colleagues (Munoz de Bustillo Lorente & de Pedraza, 2007). The pooled OR of 1.39 in our study may be regarded as moderate but, given the high prevalence of job insecurity in Europe (D'Souza et al., 2003), the public health impact of job insecurity is likely to be substantial. Considering that job insecurity will continue to increase if the current labour market trend continues (D'Souza et al., 2003), it is important to determine which factors may explain its detrimental effects on health.

It has been suggested that in the evaluation of job insecurity both the actors' individual resources and endowments (education, income, etc.) and contextual factors at the macro level (such as legislation standards, economical environment) play a role (Coleman, 1986; Erlinghagen, 2008). Similarly, factors that may explain the effects of job insecurity on health may be related to the individual and its micro-environment and to economic, societal and cultural factors at macro level.

Several individual factors which could contribute to the explanation of the investigated association were considered in our multivariate analysis. The results indicated that age, sex, marital and managerial status, work-related factors, previous chronic diseases or behavioural factors did not explain the investigated association. To what extent other factors on micro-level, such as the family or the workplace context, the economic and the social network, self-esteem or mental health, contribute to the investigated association needs to be investigated in future studies.

It was hypothesized that factors on macro level, such as the extent of labour market regulations, the degree of unionization and collective power, investments in and the strictness of employment protection may result in differences between countries in the relationship between job insecurity and poor health. However, our results indicate that there was no heterogeneity in the relationship between job insecurity and health across the European samples included in our study. The association of job insecurity with poor health was present in different countries, including several states with good welfare regimes. This seems to suggest that a well-developed welfare state does not always eliminate the negative consequences of job insecurity (Böckerman, 2002; Erlinghagen, 2008).

Our findings that the effect of an insecure job is not modified by education, age or marital status is consisted with several previous reports (Cheng et al., 2005; De Witte, 1999; McDonough, 2000; Sverke et al., 2002). On the other hand, in a recent Danish study job insecurity seemed to have a somewhat more deleterious effect among those aged <50 years than in older individuals (Rugulies et al., 2008). Previous studies investigating gender differences in the health consequences of job insecurity have yielded conflicting results. Several studies found that job insecurity was more detrimental for men than for women (Cheng et al., 2005; De Witte, 1999; Ferrie et al., 1995; Kopp, Skrabski, Székely, Stauder, & Williams, 2007; Rugulies et al., 2006), others showed the opposite (Ferrie et al., 1995; Rugulies et al., 2008), whereas a third group of studies found no evidence for the gender effect modification (McDonough, 2000; Pelfrene et al., 2003; Wang, Lesage, Schmitz, & Drapeau, 2008). We found a slightly, though not significantly increased risk of poor health associated with job insecurity among women compared to men.

A tentative explanation for these differences between studies may be that the greater expectations of the work roles for men and of the family roles for women in some countries (e.g. Spain) may result in a more distressing experience of job insecurity for men. In societies where the gender expectations of the work role differ to a smaller extent, the effect of job insecurity is more likely to be similar for the two genders or eventually to be more detrimental for women as they face more expectations regarding family roles.

### Limitations

Our study has several limitations. First, due to the cross-sectional design of the study no conclusions regarding causality can be drawn. Beside the proposed causal relationship, the health selection hypothesis, i.e. that people experiencing poor health are more prone to be offered and to accept less secure jobs, is also plausible. However, by controlling for lifetime diagnosis of chronic diseases we tried to minimize the confounding effect of previous health. Further investigations using longitudinal data are needed to provide explanations for the investigated association.

Second, the use of a single item to assess job insecurity instead of a validated questionnaire limits the accuracy of the exposure measurement. The meta-analysis conducted by Sverke et al. (2002) suggests that the use of single items to measure job insecurity compared to multi-item questionnaires is likely to result in an underestimation of the association between job insecurity and outcome. However, as all the three surveys on which our analysis was based intended to involve a large number of subjects with different socioeconomic background, the measures had to be simple and brief. Similarly the slight difference in item-formulation and answer options between the SHARE and the other two studies warrants caution when comparing country-specific job insecurity prevalence.

Third, due to the self-report of job insecurity we do not know to what extent this indicates real threat to continuity of the job or only a subjective appraisal of the situation. Thus the association between job security and health might be due to a third factor, a potential confounder such as negative affectivity or personality.

Fourth, due to the small sample size in some countries, the power might have been limited to detect significant interactions between job insecurity and the investigated demographic factors.

Finally, though restricting our analysis to subjects 45–70 years has the advantage of reducing to some extent the confounding effect of age, it limits the possibilities to generalize our findings to other age groups. It has been suggested that the effect of job insecurity may be most detrimental in younger individuals – especially among those aged 30–45 years – due to their family responsibilities, bank loans and thus a strong dependency on a steady income (De Witte 1999; Sverke et al., 2002). It is thus plausible that the inclusion of younger individuals in our study (primarily those aged 30–45 years) would have resulted in stronger associations between job insecurity and health. Findings from a Danish study indicate that job insecurity has somewhat stronger effect among those aged <50 years than in older individuals (Rugulies et al., 2008). However, as already discussed, other authors did not find evidence for effect modification from age on the association between job insecurity and health (Cheng et al., 2005; De Witte 1999; McDonough, 2000; Sverke et al., 2002). Nevertheless, given the evidence that middle-aged persons, particularly in Eastern Europe, are at an increased risk of morbidity and mortality, our study contributes to the identification of psychosocial risk factors for poor health in this high risk population.

## Conclusions

Our findings indicate that an important proportion of middle-aged individuals in Europe are affected by job insecurity and that having an insecure job is associated with an increased risk of poor health in most of the countries included in the analysis. Given that job insecurity is likely to increase as the labour market becomes more globalised, governments and labour unions need to pay attention to job insecurity and its public health consequences. Future research could further investigate individual and societal characteristics which may modify the effects of job insecurity on different health outcomes and the pathways through which an insecure job can lead to impaired health.

## Figures and Tables

**Table 1:** Characteristics of the study population.

Country	*N*	Group for age in years (%)					Male sex (%)	Job insecurity (%)	Self-rated health (%)				
		45–49	50–54	55–59	60–65	66–70			Very good	Good	Fair	Poor	Very poor
Austria	350	6.8	49.0	34.6	7.0	2.5	53.8	19.4	31.3	47.9	18.6	1.7	0.6
Belgium	909	6.6	48.3	35.2	8.8	1.1	54.2	23.1	30.5	55.9	12.3	1.3	0
Czech Republic	4003	30.8	34.7	22.9	8.7	3.0	51.9	41.0	4.9	46.9	42.4	5.4	0.4
Denmark	641	8.9	39.6	34.9	14.3	2.3	49.8	18.6	35.2	51.2	11.4	1.7	0.6
France	889	8.1	47.0	36.9	7.5	0.5	47.2	17.6	23.8	57.6	15.7	2.5	0.4
Germany	864	3.2	46.6	32.1	15.1	3.1	52.0	21.9	22.4	56.6	18.2	2.8	0
Greece	806	9.6	43.8	30.2	14.0	2.4	62.9	28.5	43.9	43.8	11.4	0.8	0.1
Hungary	1286	8.6	20.6	23.2	22.1	25.5	42.6	40.4	6.1	48.0	40.2	4.8	0.9
Israel	823	5.4	31.8	37.3	17.2	8.3	48.6	24.1	38.1	34.8	23.8	3.1	0.2
Italy	469	4.7	40.5	37.7	12.7	4.4	59.1	27.7	17.6	56.4	23.5	2.3	0.2
The Netherlands	878	5.9	42.1	40.3	10.6	1.0	55.4	31.7	28.2	57.9	13.2	0.7	0.1
Poland	4315	32.6	31.0	21.6	10.3	4.5	54.0	41.7	6.5	46.2	41.1	5.7	0.5
Russia	4953	26.0	28.8	24.3	11.6	9.4	52.3	30.8	0.2	14.0	72.5	12.8	0.4
Spain	465	3.0	42.1	35.5	16.6	2.8	57.9	14.2	19.1	58.7	18.1	3.6	0.4
Sweden	1211	2.5	32.9	37.0	24.8	2.7	46.1	19.0	42.7	36.6	18.1	2.3	0.2
Switzerland	383	4.9	39.4	30.9	19.6	5.2	53.9	19.6	44.6	46.1	8.5	0.8	0

**Table 2:** Odds ratios and 95% confidence intervals for the association between job insecurity and self-reported health.

Country	*N*	OR (95% CI)			
		Model 1	Model 2	Model 3	Model 4
Austria	350	1.61 (0.85–3.04)	1.69 (0.87–3.26)	1.57 (0.78–3.17)	1.71 (0.83–3.50)
Belgium	909	1.10 (0.70–1.73)	1.03 (0.64–1.64)	1.02 (0.64–1.64)	1.05 (0.65–1.71)
Czech Republic	4003	1.41 (1.24–1.61)	1.31 (1.15–1.50)	1.31 (1.14–1.49)	1.31 (1.14–1.49)
Denmark	641	1.99 (1.18–3.33)	1.98 (1.16–3.37)	1.84 (1.06–3.18)	1.80 (1.04–3.13)
France	889	1.45 (0.96–2.20)	1.30 (0.85–1.99)	1.22 (0.79–1.90)	1.26 (0.79–2.01)
Germany	864	2.00 (1.38–2.91)	1.96 (1.33–2.88)	2.02 (1.36–3.00)	2.00 (1.33–3.01)
Greece	806	1.54 (0.98–2.40)	1.49 (0.93–2.39)	1.62 (1.00–2.63)	1.71 (1.04–2.81)
Hungary	1286	1.66 (1.32–2.08)	1.53 (1.21–1.94)	1.55 (1.22–1.97)	1.56 (1.21–1.99)
Israel	823	1.77 (1.26–2.51)	1.63 (1.14–2.33)	1.71 (1.19–2.45)	1.65 (1.13–2.40)
Italy	469	1.56 (0.999–2.44)	1.54 (0.97–2.46)	1.56 (0.97–2.50)	1.44 (0.89–2.35)
The Netherlands	878	1.76 (1.19–2.60)	1.73 (1.15–2.60)	1.74 (1.16–2.63)	1.85 (1.22–2.80)
Poland	4315	1.41 (1.24–1.60)	1.37 (1.21–1.56)	1.38 (1.21–1.57)	1.39 (1.22–1.59)
Russia	4953	1.19 (0.99–1.42)	1.22 (1.02–1.47)	1.26 (1.04–1.51)	1.27 (1.06–1.53)
Spain	465	1.43 (0.79–2.60)	1.42 (0.77–2.60)	1.43 (0.76–2.68)	1.60 (0.84–3.04)
Sweden	1211	1.14 (0.81–1.62)	1.03 (0.71–1.49)	0.98 (0.67–1.42)	1.00 (0.68–1.47)
Switzerland	383	1.53 (0.68–3.44)	1.25 (0.53–2.91)	1.32 (0.52–3.34)	1.35 (0.53–3.45)

Model 1 includes job insecurity, age and sex. Model 2 includes job insecurity, age, sex, education, managerial status, type of work (part-time job, full-time job, working excess hours) and marital status. Model 3 includes job insecurity, age, sex, education, managerial status, type of work (part-time job, full-time job, working excess hours), marital status, physical activity, body-mass index, smoking and frequency of alcohol consumption. Model 4 includes job insecurity, age, sex, education, managerial status, work hours, type of work (part-time job, full-time job, working excess hours), physical activity, body-mass index, smoking, frequency of alcohol consumption and existence of at least a chronic disease from diabetes, cancer, stroke, hypertension, coronary heart disease.
